# The Synergy Between Deterministic and Neutral Processes Drives Turnover‐Dominated Beta Diversity of Non‐Volant Small Mammals in the Western Tianshan Mountains, China

**DOI:** 10.1002/ece3.74538

**Published:** 2026-10-30

**Authors:** Xiaohan Wang, Wenyu Song, Yawen Xing, Yalan Wang, Xiaoxin Pei, Kenneth Otieno Onditi, Hongjiao Wang, Quan Li, Xueyou Li, Zhongzheng Chen, Xuelong Jiang

**Affiliations:** ^1^ Collaborative Innovation Center of Recovery and Reconstruction of Degraded Ecosystem in Wanjiang Basin Co‐Founded by Anhui Province, School of Ecology and Environment Ministry of Education, Anhui Normal University Wuhu China; ^2^ State Key Laboratory of Genetic Evolution and Animal Models, Yunnan Key Laboratory of Biodiversity and Ecological Conservation of Gaoligong Mountain Kunming Institute of Zoology, Chinese Academy of Science Kunming China; ^3^ Vector Laboratory Institute of Pathogens and Vectors, Dali University Dali China

**Keywords:** beta diversity, community assembly, non‐volant small mammals, turnover, western Tianshan Mountains

## Abstract

Exploring multifaceted beta diversity, including its turnover and nestedness components, is crucial for elucidating the drivers of community assembly. Arid mountain ecosystems, with their strong environmental filtering and fragmented habitats, serve as a useful model system for testing how these drivers interact across multiple dimensions of diversity. Here, we compared taxonomic, functional, and phylogenetic beta diversity in non‐volant small mammal communities across environmental gradients in arid areas of the western Tianshan Mountains, China, and inferred the associated community‐assembly processes. Species turnover predominantly structured beta diversity across all three dimensions. Decomposition of multifaceted beta diversity revealed a consistent pattern: functional dissimilarity was highest, followed by taxonomic and phylogenetic dissimilarity. Hierarchical partitioning identified elevational distance as the most significant factor influencing beta diversity and its turnover component, and nestedness was driven by multiple factors. We also confirmed a significant distance–decay relationship for overall community dissimilarity and its turnover component. Moreover, analyses of community‐assembly mechanisms suggested a synergistic interaction between deterministic and neutral processes, whereby the influence of environmental filtering weakened while that of limiting similarity strengthened as elevational distance increased. Collectively, these findings clarify the patterns and drivers of beta diversity in arid montane regions and provide a practical basis for biodiversity conservation in Central Asian mountain ecosystems.

## Graphical abstract

Through a survey of small mammals in the western Tianshan Mountains, we discovered that the synergy between deterministic and neutral processes drives the turnover‐dominated beta diversity of small mammals.

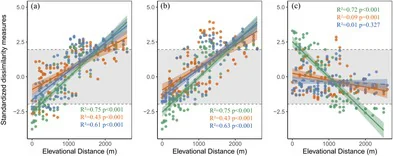

## 1 Introduction

Beta diversity, variation in community composition among sites, is a foundational concept in biodiversity research that connects local assembly processes to regional biogeographic patterns (Whittaker 1960, 1972; Hill et al. 2021). It is now standard practice to evaluate beta diversity across taxonomic, functional, and phylogenetic dimensions, as this integrative framework enables robust inference of the ecological and evolutionary mechanisms structuring communities (Faith 1992; Webb et al. 2002; Petchey and Gaston 2002; Fukami et al. 2005; Siefert et al. 2013; Mouquet et al. 2012).

The additive partitioning framework formalized by Baselga (2010) is a widely used approach to quantifying beta diversity (Baselga and Leprieur 2015). In this approach, pairwise beta diversity is decomposed into two components: turnover and nestedness. Turnover reflects species replacement between communities and is often associated with environmental differences, competition, or geographic barriers (Qian et al. 2005; Baselga 2010, 2012). Nestedness, by contrast, reflects species loss or gain, where less diverse communities form subsets of more diverse ones, a pattern often linked to selective extinction, immigration, or nested habitat structure (Baselga 2012). These components have distinct conservation implications. High turnover implies that conserving multiple, compositionally distinct areas is necessary, whereas strong nestedness suggests prioritizing species‐rich core areas when resources limit protection of peripheral sites (Baselga 2010; Gutierrez Cánovas et al. 2013). This partitioning framework has been applied across tropical forests, temperate grasslands, and even arid lowland ecosystems (Kelt et al. 1996; Ojeda et al. 2011), but its applicability to arid montane small‐mammal communities, particularly in Central Asian mountain systems, remains largely untested.

Identifying beta diversity drivers along environmental gradients is fundamental to understanding community assembly (Rafajlovi et al. 2017; Du et al. 2021). An increasingly used exploratory framework integrates niche theory and neutral theory (Rafajlovi et al. 2017; Du et al. 2021). Niche theory posits that deterministic biotic and abiotic processes, such as limiting similarity and environmental filtering, sort species from the regional pool into local communities. Limiting similarity arises through competitive exclusion, which constrains the degree of similarity among coexisting species (Wilson et al. 1987). Environmental filtering occurs when conditions along a gradient, from benign to harsh, selectively permit the establishment of species with suitable traits (Grinnell 1917; Leprieur et al. 2009). In contrast, neutral theory assumes ecological equivalence among species and emphasizes stochastic processes, including dispersal, reproduction, mortality, and immigration, in shaping community composition (Hubbell 2001). Empirical studies indicate that deterministic and stochastic processes frequently interact to generate beta‐diversity patterns, with their relative importance varying across spatial scales, environmental gradients, and taxonomic groups (Kraft et al. 2011; Du et al. 2021; Wang et al. 2022). For example, neutral processes may become more influential at broader spatial scales as the strength of local species interactions diminishes (Mod et al. 2020). Taxa also differ in sensitivity to these processes, reflecting differences in evolutionary histories and functional traits (Swenson et al. 2011; Weinstein et al. 2014; Du et al. 2021). Given this context dependence, no single theory fully explains community assembly across systems (Anderson et al. 2011). Quantifying the ecological processes underlying multifaceted beta diversity is therefore essential for resolving biogeographic patterns (Du et al. 2021; Wang et al. 2022) and for informing multi‐scale conservation strategies (Socolar et al. 2016; Xu et al. 2012). Niche theory posits that environmental filtering and limiting similarity deterministically sort species from the regional pool into local communities (Grinnell 1917; Wilson et al. 1987; Leprieur et al. 2009). Neutral theory, by contrast, assumes ecological equivalence among species and emphasizes stochastic dispersal, drift, and colonization dynamics (Hubbell 2001). Empirical evidence increasingly shows that the two classes of process operate concurrently, with their relative importance shifting across spatial scales, habitat types, and taxonomic groups (Kraft et al. 2011; Du et al. 2021; Wang et al. 2022).

Mountain ecosystems are widely recognized as priorities for biodiversity conservation because they support high endemism and numerous threatened species within limited geographic areas (Rahbek et al. 2019; Li et al. 2024). The steep environmental gradients characteristic of mountains, including temperature, precipitation, and elevation over short distances, make them powerful natural laboratories for studying community assembly (Sanders and Rahbek 2012). In contrast to latitudinal gradients, elevational gradients can involve distinct interactions between environmental filtering and dispersal limitation; the compressed spatial scales in mountains may reduce the influence of dispersal limitation (Wang et al. 2022). A global meta‐analysis of rodent elevational diversity further confirmed that arid and humid mountains follow fundamentally different diversity structuring rules: arid mountains exhibit increasing functional and phylogenetic dispersion with elevation, driven by strong aridity filtering at low elevations and habitat specialization at high elevations (Kohli et al. 2022). While these findings establish a clear theoretical framework for arid montane community assembly, whether these patterns extend to the temperate arid mountains of Central Asia remains largely untested.

Non‐volant small mammals (hereafter referred to as small mammals) primarily include orders such as Rodentia, Eulipotyphla, and Lagomorpha, and are important functional components of terrestrial ecosystems (Wilson and Reeder 2005; McCaffery et al. 2020). These taxa typically have limited dispersal capacity and are highly sensitive to environmental change (Umetsu et al. 2008; Gomes et al. 2011). Their communities often exhibit pronounced turnover along environmental gradients (Varzinczak et al. 2019; Du et al. 2021), making them informative systems for studying biodiversity‐assembly mechanisms (Moritz et al. 2008; Moorhead et al. 2017). Previous studies in arid mountains have documented pronounced species turnover of small mammals along elevational gradients, with environmental filtering consistently identified as a dominant driver (Melo et al. 2009; Andrade and Monjeau 2014). However, most of these studies are restricted to taxonomic beta diversity; integrative analyses combining functional and phylogenetic dimensions remain scarce, especially in arid mountains of Central Asia. The environmental and biological characteristics of arid montane systems suggest that taxonomic, functional, and phylogenetic beta diversity may not respond identically (Kohli et al. 2022; Du et al. 2021). Steep elevational gradients and restricted species ranges may promote taxonomic turnover (Andrade and Monjeau 2014). The extent of functional turnover should depend on whether the replacing species differ in body size and locomotor strategy; if replacement occurs mainly among closely related taxa with similar ecological traits, functional and phylogenetic turnover may be weaker than taxonomic turnover (Du et al. 2021).

To help address this gap, we focus on the Tianshan Mountains, a vast arid‐region mountain system that provides a pronounced environmental gradient ideal for disentangling these assembly processes. The Tianshan Mountains, located in the heart of Eurasia, constitute a major mountain range and biodiversity hotspot, forming the largest mountain system in the arid temperate zone. The range is surrounded by deserts, bounded by the Taklamakan Desert to the south and the Gurbantünggüt Desert to the north, creating an “island‐like” montane system within an arid matrix (Xu et al. 2012). Despite its biogeographic distinctiveness, biodiversity research in this region has focused predominantly on plants and soil microbes (Bai et al. 2006; Zhang et al. 2012; Jiang et al. 2014; Wu et al. 2015; Cheng et al. 2020). For small mammals, existing work is limited to faunal surveys, species distribution records, and single‐species genetic studies (Asakawa et al. 2001), with no comprehensive analysis of community‐level beta diversity across taxonomic, functional, and phylogenetic dimensions. As a result, the assembly mechanisms structuring small‐mammal communities in this iconic Central Asian arid mountain system remain entirely unresolved.

In this study, we investigated small mammal communities in mountainous desert ecosystems of the western Tianshan Mountains to clarify the distribution patterns and drivers of small mammal beta diversity in Central Asia's arid region. Specifically, we aimed to: (1) characterize taxonomic, functional, and phylogenetic differentiation in small mammal communities by comparing total beta diversity and its turnover and nestedness components across these dimensions; (2) evaluate relationships between environmental predictors and beta‐diversity variation using distance‐based regression; (3) test the distance–decay model by assessing linear relationships between elevational distance and beta diversity; and (4) apply null models to quantify the relative contributions of niche‐based and neutral processes to community assembly.

## 2 Methods

### 2.1 Study Area

The study was conducted in the western Tianshan Mountains, Xinjiang Uygur Autonomous Region, China (42°27′–45°07′ N, 80°32′–86°01′ E), spanning five areas that differ in climate, elevation, and habitat type (Table S1): Kurdening (Gongliu County), Shata (Zhaosu County), Tukai Desert (Huocheng County), Ebinur Lake (Jinghe County), and Moso Bay Desert (Manas County) (Figure 1).

**FIGURE 1:**
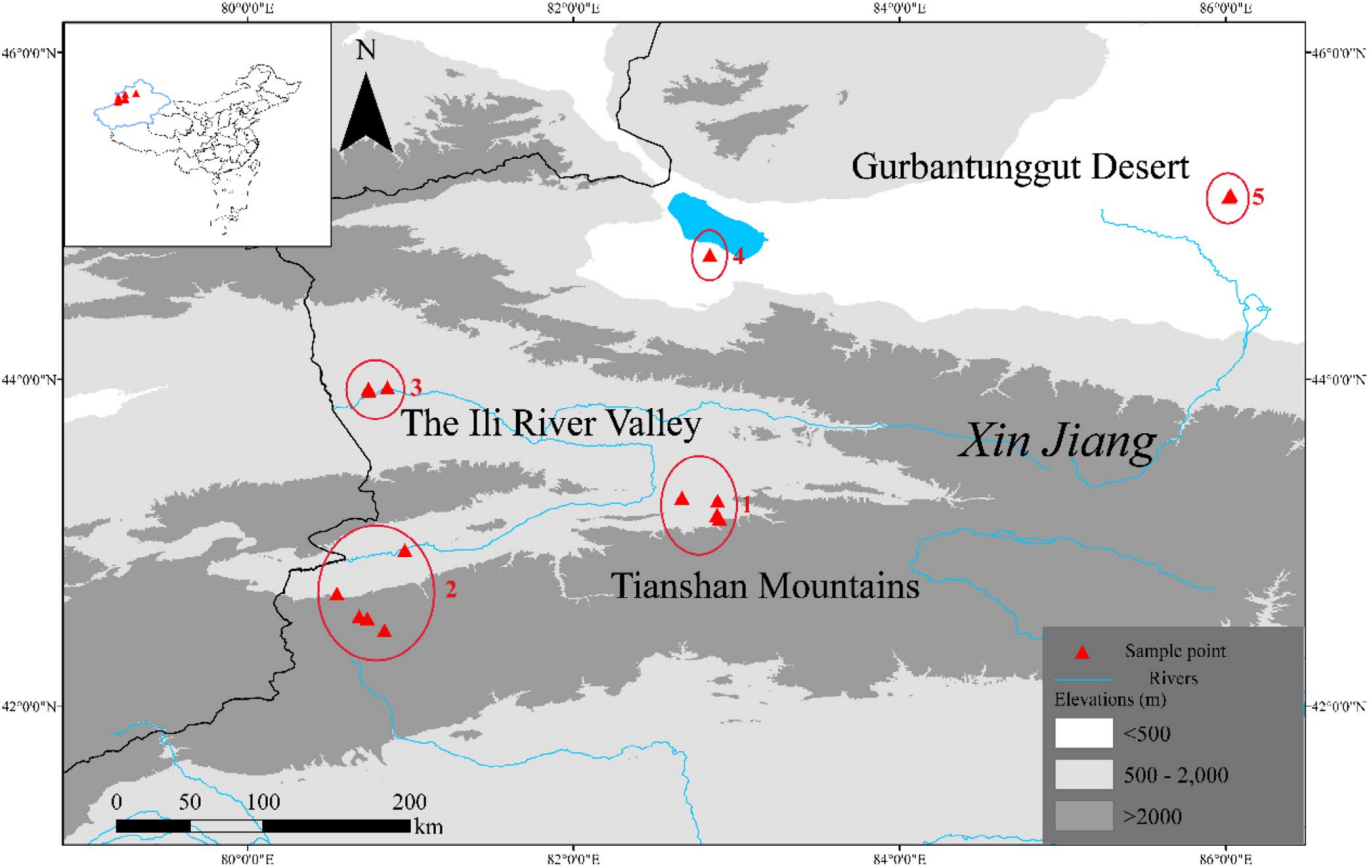
Distribution map of sampling plots in the western Tianshan Mountains, China. Area 1: Kurdening, Gongliu County; Area 2: Shata, Zhaosu County; Area 3: Tukai Desert, Huocheng County; Area 4: Ebinur Lake, Jinghe County; Area 5: Moso Bay Desert, Manas County. Additional site information is provided in Table S1.

Climatic conditions also vary among areas. Kurdening receives an average annual precipitation of 223 mm and has an annual mean temperature of 4.8°C, representing a semi‐arid to arid transition zone. Shata receives 323 mm of annual precipitation and has an annual mean temperature of 0.9°C; it is among the wettest localities on the northern slopes of the Tianshan Mountains. Tukai Desert (226 mm; 9.5°C) exhibits a temperate desert climate. Ebinur Lake receives 218 mm annually and has an annual mean temperature of 9.8°C. Moso Bay Desert is the driest site (134 mm; 8.4°C) and is characterized by extremely arid conditions.

Vegetation in the western Tianshan Mountains transitions from temperate desert at low elevations to mountain steppe, montane coniferous forest, alpine meadow, alpine cushion vegetation, and, at the highest elevations, a nival zone, forming a complete sequence of vertical vegetation belts (Liu 2017). These habitats support diverse flora and fauna, making the Tianshan Mountains an important refugium for endemic species in arid Central Asia (Wang et al. 2011; Xu et al. 2012; Zhang et al. 2021).

### 2.2 Community Data

#### 2.2.1 Field Sampling and Community Matrix

Field surveys were conducted from June to July 2021 along elevational gradients in the western Tianshan Mountains. Fifteen transects were established between 200 and 2800 m a.s.l. Each transect comprised 30 sampling points spaced at approximately 10 m intervals, for a total of 450 sampling points. Small mammals were sampled using standard trap‐night methods, including Sherman live traps baited with oatmeal, snap traps baited with raw peanuts, and pitfall traps constructed from plastic buckets. At each sampling point, one live trap and one snap trap were placed 1–2 m apart. Pitfall traps were positioned along likely insectivore runways within transects.

All traps were deployed for two consecutive nights per transect. Traps were checked and rebaited on the morning of the second day and were relocated to a new transect on the morning of the third day. The elevational range of each species was defined as the minimum and maximum elevations at which individuals were captured.

#### 2.2.2 Phylogenetic Data and Reconstruction

Complete mitochondrial genome sequences for all sampled species were obtained from newly generated next‐generation sequencing data and from GenBank. To reconstruct phylogenetic relationships, we first used PartitionFinder2 to identify optimal partitioning schemes and substitution models under the Bayesian Information Criterion (BIC) (Lanfear et al. 2017). Phylogenetic inference was then conducted in BEAST v2.6.7 (Bouckaert et al. 2014) using concatenated sequences from 13 mitochondrial protein‐coding genes and two rRNA genes. Markov chain Monte Carlo (MCMC) analyses were run for 300 million generations, sampling every 10,000 generations. Convergence and sampling adequacy were assessed in Tracer v1.7 (Rambaut et al. 2018); all parameters achieved effective sample sizes (ESS) > 200 after discarding the first 10% of samples as burn‐in. The maximum clade credibility tree was generated in TreeAnnotator v2.6.7 and visualized in FigTree v1.4.0 (Figure S1).

#### 2.2.3 Functional Trait Data and Functional Space

We selected five morphological traits (body mass, head‐body length, tail length, hind foot length, and ear length) that are widely adopted in functional diversity studies of small non‐volant mammals, as they effectively capture key axes of species' ecological strategies along body size, locomotion, and habitat adaptation dimensions. Morphological traits are fundamental components of the functional trait framework, which can influence individual fitness through effects on growth, reproduction, and survival (Violle et al. 2007).

Specifically, body mass and head‐body length characterize overall body size, which is tightly linked to metabolic rate, resource acquisition capacity, and trophic niche position, and shows clear adaptive variation along climatic and elevational gradients in arid mountains (Searing et al. 2023). Tail length is associated with balance ability and the continuum of arboreal to terrestrial habitat use, with longer tails supporting more complex three‐dimensional habitat navigation (Hager and Hoekstra 2021). Hind foot length reflects locomotor capacity and substrate preference, corresponding to differentiation among rocky, meadow, and shrub habitats across elevations (Lövy et al. 2024). Ear length correlates with auditory sensitivity for predator detection and thermoregulatory adaptation to arid environments, which is of particular ecological relevance in our study system (Alhajeri and Steppan 2018).

Five morphological traits were measured for up to 10 individuals per species; when fewer than 10 individuals were captured, all available specimens were measured. Body mass was recorded in grams (g), and all four linear traits were recorded in millimeters (mm). Trait values were summarized to construct a species × trait matrix for functional analyses. A Gower distance matrix was computed from the continuous trait data, and functional space was constructed using Principal Coordinates Analysis (PCoA). The first two PCoA axes explained 90.9% of total inertia and were retained to represent functional space (Tables S3 and S4).

Phylogenetic signal in the five traits was assessed using Blomberg's K (Blomberg et al. 2003) with the multiPhylosignal function in the R package picante (Kembel et al. 2010), based on 1000 randomizations (Table S3).

### 2.3 Environmental Predictors

Geometric constraints, climatic gradients, habitat characteristics, anthropogenic disturbance, and productivity regimes can jointly shape species distributions across spatial scales (Sauvajot et al. 1998; Rahbek 2005; McCain 2010; Sanders and Rahbek 2012; Carmignotto et al. 2022). We initially screened seven predictors: annual mean temperature (AMT), annual precipitation (AP), productivity (NDVI), habitat heterogeneity (HH), human disturbance (HD), elevational distance (ELE), and geographic distance (GEO). VIFs were calculated using car::vif (Fox and Weisberg 2018). AMT had VIF > 10 and was removed before model fitting; the six retained predictors (AP, NDVI, HH, HD, ELE, and GEO) all had VIF < 5 (Table S10).

AP was obtained from WorldClim v2.1 at 30 arc‐second resolution (Fick and Hijmans 2017). NDVI was derived from the “1990–2021 China 30 m Annual Maximum NDVI Dataset” (Cao et al. 2023) and used as a proxy for primary productivity. Habitat heterogeneity (HH) was quantified using the Shannon diversity index in FRAGSTATS (McGarigal 1995) based on the “1985–2022 China 30 m Annual Land Cover Dataset” (Yang and Huang 2024). Human disturbance (HD) was represented by population density from WorldPop. Geographic distances (GEO) among sites were calculated using the distm function in the R package geosphere. Elevational distance was defined as the absolute difference in elevation between pairs of sampling sites. Climatic, productivity, habitat, and anthropogenic variables were treated as environmental predictors, whereas GEO and ELE were retained as spatial distance matrices. ELE was interpreted as composite separation along the gradient rather than as a direct measure of dispersal limitation.

### 2.4 Observed and Standardized Beta‐Diversity Metrics

Observed taxonomic, phylogenetic, and functional beta diversity was calculated for both multiple‐site and pairwise communities using Sørensen dissimilarity based on species presence–absence. Total beta diversity was partitioned into turnover and nestedness components following Baselga's framework (Baselga 2010). Functional beta diversity was computed using trait‐derived dissimilarities (Gower‐based functional distances represented in PCoA space), and phylogenetic beta diversity was computed from the BEAST‐derived phylogeny (branch‐length‐based dissimilarity). Beta‐diversity calculations and their turnover/nestedness decompositions were implemented in the R package betapart (Baselga and Orme 2012).

Null‐model analyses were used to infer community assembly processes by comparing observed beta diversity against expectations under random community assembly (Swenson and Enquist 2009). We generated 999 randomized community matrices using the “independentswap” algorithm (Gotelli 2000), which preserves species occurrence frequencies and site species richness (Song et al. 2020). Standardized effect sizes (SES) were calculated as: SES = (Observed − Mean_null_)/SD_null_. Values of SES > 1.96 or SES < −1.96 were interpreted as significant deviations from the null expectation. Positive deviations (SES > 1.96) were interpreted as overdispersion consistent with limiting similarity, whereas negative deviations (SES < −1.96) were interpreted as underdispersion consistent with habitat filtering.

### 2.5 Statistical Analyses

Mantel tests (9999 permutations; mantel function in the R package vegan) were used to assess correlations between beta‐diversity dissimilarity matrices and matrices of environmental distance (Oksanen et al. 2013), statistical details are provided in Table S2. Linear regression (lm in stats) was used to evaluate relationships between elevational distance and community dissimilarity (distance–decay). Multiple regression on distance matrices (MRM; 9999 permutations; MRM function in ecodist) was used to quantify the joint effects of environmental distances on beta diversity (Goslee and Urban 2007). Predictor distance matrices were standardized using the stdize function in the R package MuMIn prior to MRM analyses. Hierarchical partitioning (hier.part) was used to estimate the independent contribution of each predictor. Ordinary least squares regression was used to model relationships between standardized beta diversity (SES) and elevational distance. All analyses were conducted in R v4.3.3.

## 3 Results

### 3.1 Sampling Composition and Overview

Our field survey captured a total of 610 individual non‐volant small mammals (Table S5). This comprehensive dataset comprised 20 species across 15 genera, 8 families, and 3 orders (Rodentia, Eulipotyphla, and Lagomorpha). Sampling was conducted at 15 distinct sites (Table S1) stratified along extensive environmental gradients, ranging from the arid lowlands at 200 m to the high‐altitude zones at 2800 m above sea level in the western Tianshan Mountains.

The captured taxa represented a wide spectrum of the regional small mammal fauna. The order Rodentia was the most diverse and abundant, constituting the majority of captures. Within Rodentia, the family Cricetidae was particularly prominent, featuring key species such as *Apodemus uralensis* (27.70% of total captures) and *Myodes centralis* (14.59%). Species from the families Muridae (e.g., *Meriones meridianus* , 14.10%) and Dipodidae were also well represented, especially at lower elevations. The order Eulipotyphla, represented by two species of shrews ( *Sorex asper* and *Sorex minutus* ), was primarily captured in higher elevation sites. A single individual of the Lagomorpha, *Ochotona macrotis* , was recorded at the highest sampling point.

The distribution of species across the elevational gradient revealed clear turnover patterns, with many species exhibiting restricted elevational ranges (Table S7). For instance, desert‐adapted species like *Rhombomys opimus* and *Meriones libycus* were confined to low‐elevation sites (300–550 m), while specialists such as *Alticola argentatus* were found only at high elevations (2500–2800 m). In contrast, a few generalist species, notably *Apodemus uralensis* , demonstrated a broad distribution across a wide range of elevations. Furthermore, the site‐wise abundance data (Table S6) highlighted spatial variations in species composition. For example, the Kurdening site had the highest capture total (230 individuals), dominated by forest species, whereas lowland sites like Moso Bay Desert were characterized by desert‐adapted species. This structured distribution along the gradient underscores the strong environmental filtering effect on community composition. The following analyses of multifaceted beta diversity and the inferred assembly mechanisms are grounded in this detailed specimen collection.

### 3.2 Multifaceted Beta Diversity: Components and Comparisons

Across the three dimensions, multiple‐site beta diversity was highest for the functional dimension (0.885), followed by the taxonomic (0.817) and phylogenetic dimensions (0.761). In all cases, turnover was the dominant component of beta diversity (Table 1). Turnover accounted for the largest proportion of taxonomic beta diversity (93.8%, *β*sim = 0.766), followed by phylogenetic beta diversity (89.6%, *β*sim = 0.682) and functional beta diversity (82.8%, *β*sim = 0.733). In contrast, functional beta diversity exhibited a comparatively strong nestedness component (*β*sne = 0.152), substantially exceeding nestedness in the taxonomic (0.052) and phylogenetic (0.079) dimensions (Table 1). Together, these results indicate pronounced cross‐dimensional differences in both the magnitude of beta diversity and the relative contributions of turnover and nestedness.

**TABLE 1:** Proportions of multifaceted beta‐diversity components at multiple sites in the western Tianshan Mountains.

Dimension	Turnover	Nestedness	Total dissimilarity	Beta.RATIO (%)
Taxonomic	0.766	0.052	0.817	93.8
Functional	0.733	0.152	0.885	82.8
Phylogenetic	0.682	0.079	0.761	89.6

*Note:* Beta.RATIO denotes the ratio of turnover to total dissimilarity.

Consistent with the multiple‐site patterns, pairwise community dissimilarities showed the same rank ordering across dimensions for total dissimilarity, turnover, and nestedness. Specifically, functional dissimilarity was consistently greater than taxonomic dissimilarity, which in turn exceeded phylogenetic dissimilarity (β‐Funcsor > β‐Taxsor > β‐Phylosor; β‐Funcsim > β‐Taxsim > β‐Phylosim; β‐Funcsne > β‐Taxsne > β‐Phylosne) (Figure 2).

**FIGURE 2:**
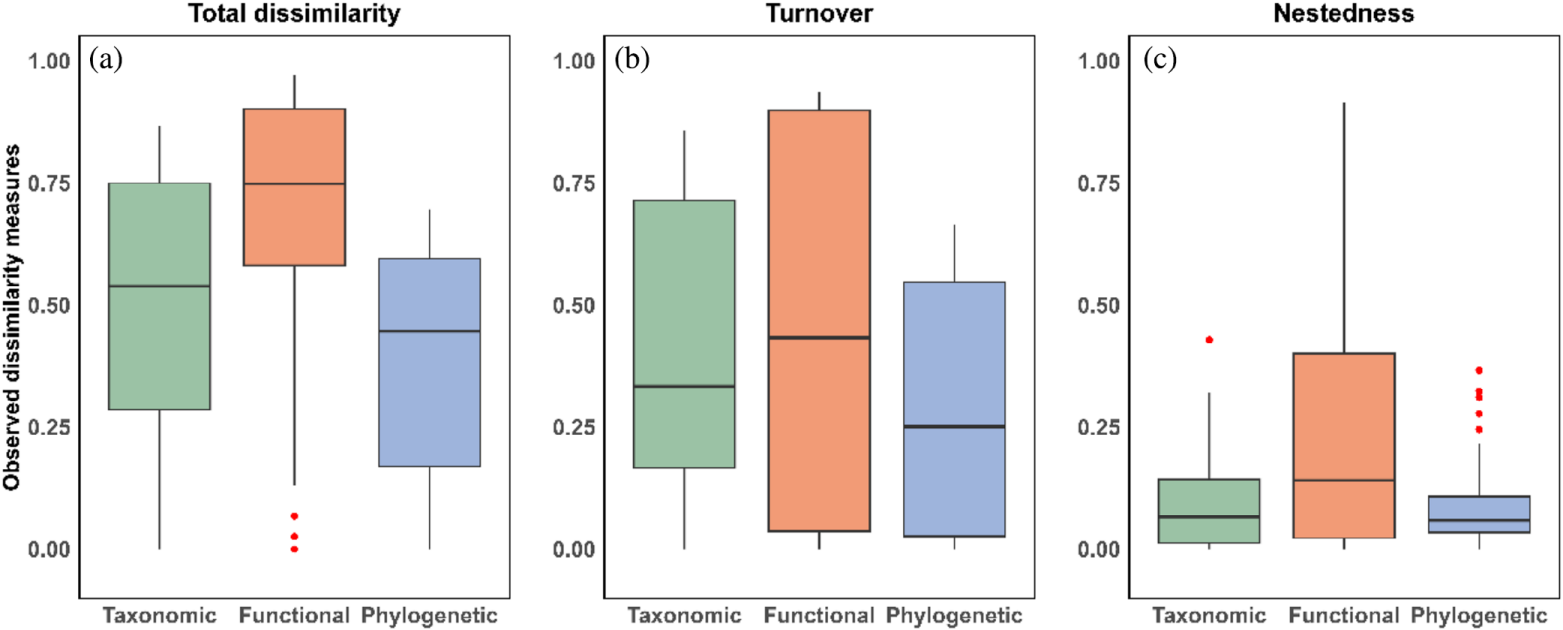
Multifaceted pairwise dissimilarities and their components (a–c). Beta diversity is partitioned into (a) total dissimilarity, (b) turnover, and (c) nestedness. Dimensions are shown as taxonomic (green), functional (orange), and phylogenetic (blue). In each panel, the median is shown as a solid line; box boundaries represent quartiles; whiskers indicate the upper and lower limits; and red points indicate outliers.

### 3.3 Elevational Patterns of Observed Beta Diversity

Elevational distance significantly influenced small mammal beta diversity in the western Tianshan Mountains. Across all three dimensions, both total dissimilarity (Figure 3a) and the turnover component (Figure 3b) were positively correlated with elevational distance. By contrast, nestedness was negatively correlated with elevational distance in the taxonomic and functional dimensions, whereas this relationship was not significant in the phylogenetic dimension (Figure 3c). Overall, total beta diversity and turnover exhibited significant distance–decay relationships across dimensions, indicating decreasing community similarity with increasing elevational separation.

**FIGURE 3:**
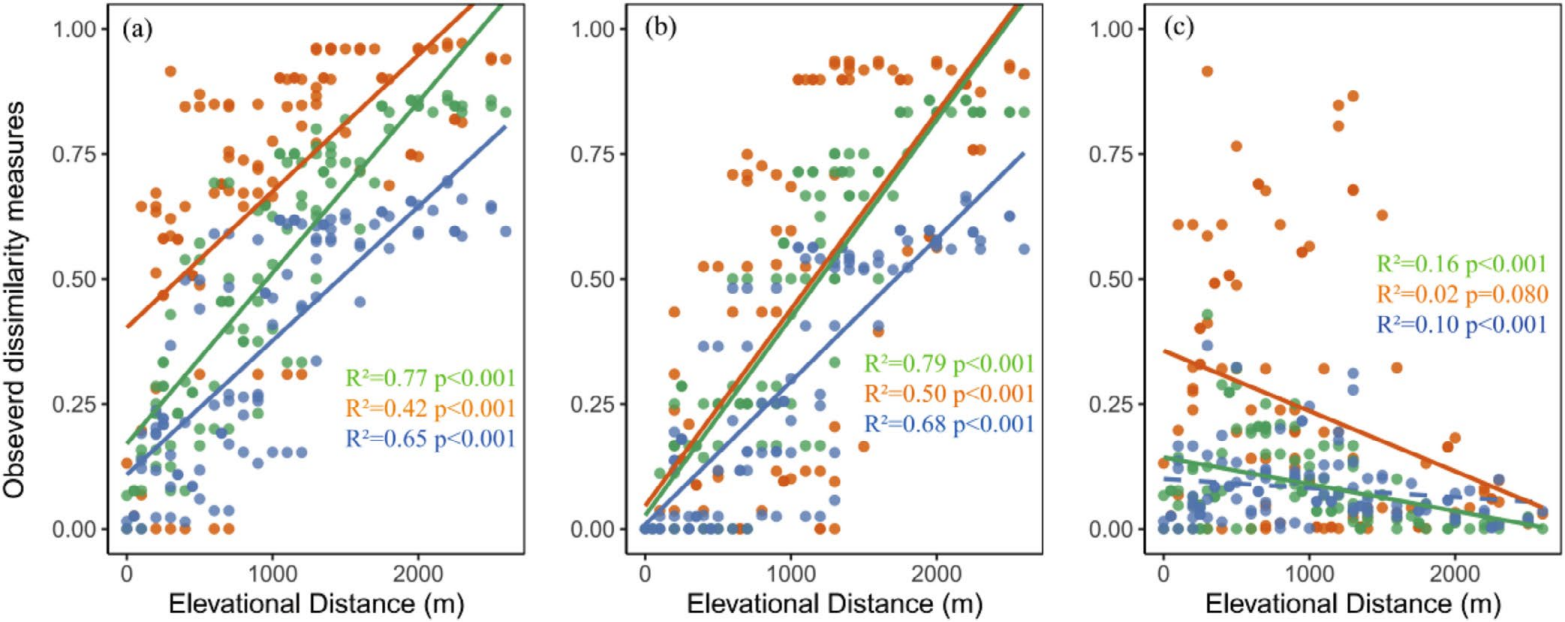
Relationships between beta diversity and elevational distance (a–c). Components are (a) total dissimilarity, (b) turnover, and (c) nestedness. Dimensions are shown as taxonomic (green), functional (orange), and phylogenetic (blue). Solid lines indicate significant relationships (*p* < 0.05), and dashed lines indicate non‐significant relationships (*p* > 0.05).

### 3.4 Contributions of Environmental and Spatial Predictors

Multiple regression on distance matrices (MRM) showed strong explanatory power for total beta diversity and turnover across taxonomic, functional, and phylogenetic dimensions. For nestedness, the taxonomic model was significant (*R* ^2^ = 0.281, *p* = 0.024), whereas the functional and phylogenetic models were not significant (Table 2).

**TABLE 2:** Effects of predictors on turnover, nestedness, and total dissimilarity across multifaceted beta diversity.

Dimensions	Components	AP	NDVI	HD	HH	GEO	ELE	*R* ^2^	*p*
Taxonomic	Total dissimilarity	−0.047*	0.046**	0.034**	0.001	0.040**	0.238**	0.832	< 0.05
	Turnover	−0.024	0.052**	0.008	0.020	0.027	0.257**	0.826	< 0.05
	Nestedness	−0.024	−0.007	0.256	−0.003	0.013	0.019	0.281	< 0.05
Functional	Total dissimilarity	−0.022	0.069**	0.042**	−0.316	0.058**	0.150**	0.526	< 0.05
	Turnover	0.040	0.067**	0.023	0.017	0.015	0.209**	0.550	< 0.05
	Nestedness	−0.616	0.002	0.187	−0.049	0.043	−0.060	0.167	0.207
Phylogenetic	Total dissimilarity	−0.012	0.043**	0.019*	−0.007	−0.001	0.172**	0.686	< 0.05
	Turnover	0.007	0.039**	0.013	0.004	0.006	0.174**	0.711	< 0.05
	Nestedness	−0.019	0.0043	0.054	−0.011	0.004	−0.002	0.079	0.574

Abbreviations: AP, annual precipitation; ELE, elevational distance; GEO, geographic distance; HD, human disturbance; HH, habitat heterogeneity; NDVI, normalized difference vegetation index.

** *p* < 0.05.

* *p* < 0.1.

For the taxonomic dimension, the MRM model explained 83.2% of the variation in total dissimilarity and 82.6% of the variation in turnover (*p* < 0.01 for both). For the functional dimension, the model explained 52.6% and 55.0% of the variation in total dissimilarity and turnover, respectively (*p* < 0.01 for both). For the phylogenetic dimension, the model explained 68.6% of the variation in total beta diversity and 71.1% of the variation in turnover (*p* < 0.01 for both). In contrast, models for nestedness across all three dimensions explained relatively little variation and were overall non‐significant.

Inspection of the directions of significant MRM coefficients indicated that AP exerted negative effects on several components. NDVI, habitat heterogeneity (HH), geographic distance (GEO), and elevational distance (ELE) exhibited positive effects on different components, and their combined influence contributed to the observed multifaceted beta‐diversity patterns.

Hierarchical partitioning further identified ELE as the most influential predictor of small mammal beta diversity in the western Tianshan Mountains (Figure 4). Across all three dimensions, total dissimilarity and turnover were explained primarily by ELE. Taxonomic nestedness was jointly associated with AP, GEO, and ELE. Functional nestedness was mainly related to AP and ELE, whereas phylogenetic nestedness was explained primarily by AP.

**FIGURE 4:**
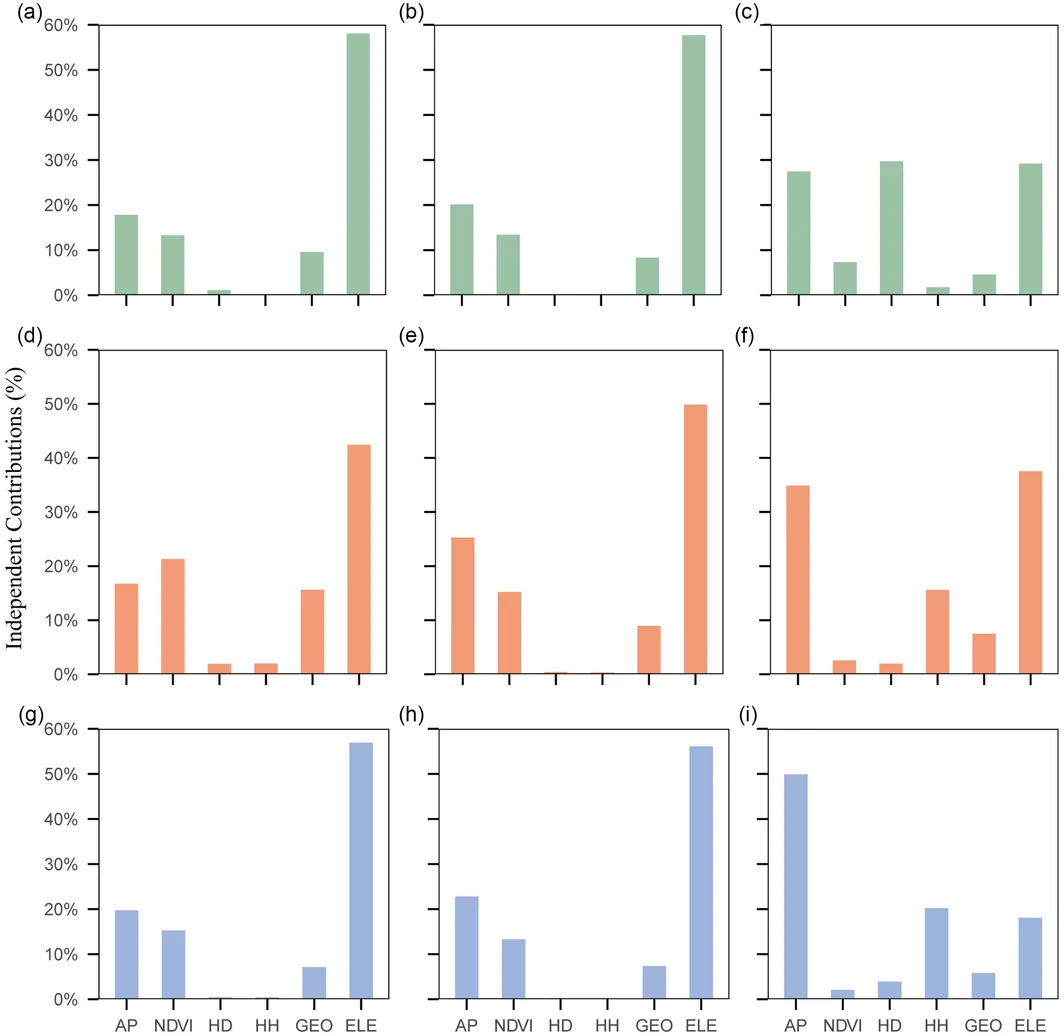
Independent contributions of each explanatory distance matrix to variation in multifaceted beta diversity: (a–c) taxonomic, (d–f) functional, and (g–i) phylogenetic beta diversity. AP, annual precipitation; ELE, elevational distance; GEO, geographic distance; HD, human disturbance; HH, habitat heterogeneity; NDVI, normalized difference vegetation index. Details are provided in Table S9.

### 3.5 Elevational Patterns of Standardized Beta Diversity and Community Assembly

Standardized effect sizes (SES) of multifaceted beta diversity were significantly associated with elevational distance for all components except phylogenetic nestedness (*p* = 0.32) (Table S9; Figure 5). For all three dimensions, SES values for total dissimilarity and turnover increased monotonically along the elevational‐distance gradient, shifting from predominantly negative values to predominantly positive values (Figure 5a,b). In contrast, SES values for taxonomic and functional nestedness decreased monotonically from above zero to below zero with increasing elevational distance (Figure 5c). For phylogenetic nestedness, most SES values remained within the null expectation bounds (−1.96 to 1.96) across the elevational gradient (Figure 5c).

**FIGURE 5:**
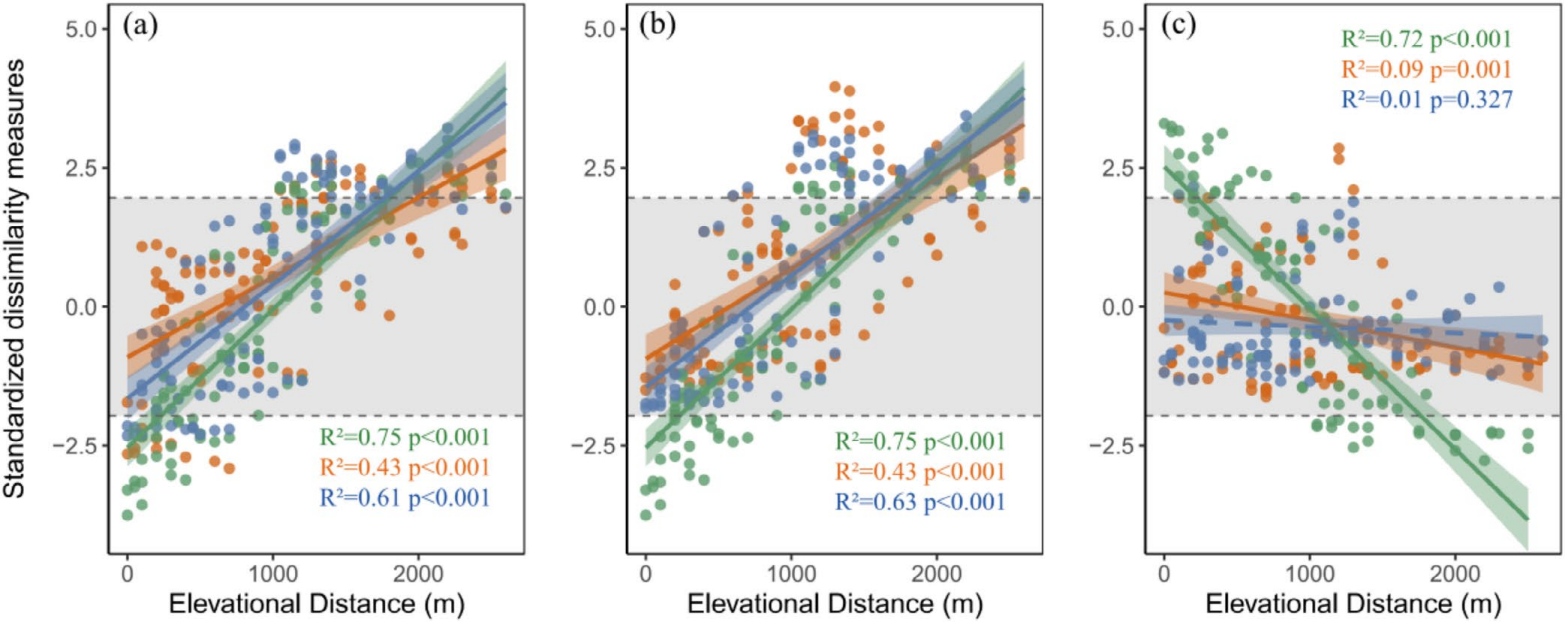
Standardized effect sizes (SES) of multifaceted beta diversity for small mammal communities along the elevational gradient. Shaded bands indicate the null expectation range (−1.96 to 1.96). Solid lines show fitted elevational trends. Additional results are provided in Table S8.

## 4 Discussion

In this study, we applied a beta diversity analytical framework, incorporating the Sørensen index and Baselga's additive partitioning, to examine taxonomic, functional, and phylogenetic beta diversity in small mammal communities along a 200–2800 m elevational gradient in the western Tianshan Mountains. We compared the effects of environmental variables on these diversity metrics. Species turnover represented the dominant component of beta diversity in all dimensions. Functional dissimilarity was greater than both taxonomic and phylogenetic dissimilarity. Elevational distance emerged as the most influential predictor of beta diversity. A significant distance–decay relationship was observed for both overall community dissimilarity and its turnover component. Both deterministic and neutral processes shaped community structure. These findings enhance understanding of the structure and assembly mechanisms of small mammal communities across environmental gradients in the western Tianshan Mountains and provide guidance for the conservation of these communities in arid mountain regions.

### 4.1 Multifaceted Beta‐Diversity Patterns

Intercommunity dissimilarity is shaped by two distinct processes: species turnover and species loss/gain (nestedness) (Baselga 2010). Turnover reflects replacement of species among communities, whereas species loss or gain generates differences in richness that can produce nested patterns (Lennon et al. 2001). Numerous studies have shown that environmental heterogeneity can drive pronounced differences in community composition (Du et al. 2021; Wang et al. 2022). Against this background, we partitioned multi‐site and pairwise taxonomic, functional, and phylogenetic beta diversity of small mammals in the arid western Tianshan Mountains into total dissimilarity, turnover, and nestedness components.

Our multi‐site results indicate that turnover dominates compositional differentiation across all three dimensions, whereas nestedness contributes comparatively little to overall dissimilarity (Figure 2). This pattern implies that spatial species replacement is the primary process structuring differences among small mammal communities (Baselga 2010), consistent with recent findings in rodents (Du et al. 2021) and insectivores (Ke et al. 2023). Dominance of turnover has been widely documented across taxa and regions (Bishop et al. 2015; Foord and Dippenaar Schoeman 2016; Wang et al. 2022; Ding et al. 2024; Wen et al. 2024), and our results reinforce this generality in an arid montane context.

Because turnover and nestedness correspond to different conservation implications, their partitioning is not merely descriptive. Predominant nestedness would suggest prioritizing species‐rich hotspots, whereas dominant turnover implies high complementarity among sites and therefore supports conserving multiple areas to maintain regional diversity (Baselga 2010; Gutierrez Cánovas et al. 2013). Given the strong contribution of turnover in the western Tianshan Mountains, effective conservation will likely require coordinated protection across multiple sites rather than reliance on a limited set of presumed “core” localities.

Across pairwise comparisons, functional dissimilarity consistently exceeded taxonomic dissimilarity, and phylogenetic dissimilarity was consistently lowest (Figure 2). This pattern differs from reports for amphibians on Mount Emei (Wang et al. 2022) and rodents in Tibet (Du et al. 2021). One plausible explanation is that species replacements in the western Tianshan Mountains occur frequently among closely related taxa, which would maintain a comparatively stable phylogenetic structure while permitting substantial functional differentiation among communities. Low phylogenetic dissimilarity could therefore reflect phylogenetic conservatism (Buckley et al. 2010; Cooper et al. 2011), whereby turnover is concentrated within lineages rather than involving substitutions among distantly related clades.

Notably, our results indicate concurrently high functional turnover and relatively high functional nestedness (β‐Funcsim > β‐Phylosim/β‐Taxsim; β‐Funcsne > β‐Phylo‐sne/β‐Taxsne), a pattern that contrasts with several earlier studies (Du et al. 2021; Wang et al. 2022; Ding et al. 2024). Given the steep environmental gradients spanning deserts to spruce forests in the western Tianshan Mountains (Table S1), high functional turnover is consistent with strong trait‐based species sorting along environmental gradients (Siefert et al. 2013; Anderson et al. 2014; Wen et al. 2024). At the same time, the substantially larger nestedness component in the functional dimension relative to the taxonomic and phylogenetic dimensions suggests disproportionate loss (or restricted occurrence) of particular functional trait combinations along environmental gradients (Du et al. 2021). This pattern is compatible with the presence of functional redundancy, in which closely related species may perform similar ecological roles such that species loss does not translate into proportional loss of functional space (Kamilar and Cooper 2013; Zhao et al. 2021; Ding et al. 2024). Our functional trait analysis is restricted to morphological metrics and does not cover ecological traits such as diet and burrowing behavior, meaning the observed functional beta diversity may only partially reflect the full multidimensional functional differentiation of small mammal communities.

Finally, regional geological history may have contributed to present‐day assembly patterns. In the western Tianshan Mountains, Paleozoic accretionary and collisional tectonic events have been proposed to influence species exchange and dispersal pathways (Xiao et al. 2013; Du et al. 2021). Taken together, these results suggest that small mammal community assembly in the western Tianshan Mountains likely reflects the combined influence of dispersal limitation and environmental filtering.

### 4.2 Key Drivers: Environmental Filtering and Spatial Distance

Small mammals are highly sensitive to environmental variation and can exhibit pronounced physiological and behavioral responses across environmental conditions (Umetsu et al. 2008; Gomes et al. 2011; Camargo et al. 2018; Varzinczak et al. 2019; Monadjem et al. 2023). Consistent with this expectation, hierarchical partitioning identified elevational distance as the most influential predictor of multifaceted total dissimilarity and turnover in the western Tianshan Mountains (Figure 4; Table 2). This aligns with patterns reported for mountain systems more broadly (Tu et al. 2017; Du et al. 2021; Wang et al. 2022) and supports central roles for environmental filtering and spatial constraints in structuring regional beta diversity.

Elevational gradients encapsulate pronounced shifts in temperature, precipitation, and habitat structure, and these climatic and environmental changes exert fundamental constraints on organismal physiology (Merriam 1894) and can influence both energetic balance and foraging behavior in small mammals (Dearing et al. 2008; Krasnov et al. 2008). Niche differentiation along elevational gradients can therefore accelerate species turnover (McCain 2010), consistent with our observed turnover‐dominated patterns across taxonomic, functional, and phylogenetic dimensions. Moreover, the strong contribution of elevational distance to turnover likely reflects the combined effects of geographic isolation and environmental heterogeneity. Relative to highly mobile taxa, small mammals are often more constrained by topography and dispersal barriers, which can elevate turnover among local communities and promote genetic differentiation and niche specialization (da Silva et al. 2014; Song et al. 2020; Wang et al. 2022; Monadjem et al. 2023).

### 4.3 Distance–Decay of Similarity

We evaluated relationships between elevational distance and multifaceted beta diversity (Figure 3). Elevational distance, as a composite measure of environmental and spatial separation along the gradient, can shape community composition (Du et al. 2021). Across all three dimensions, total dissimilarity and turnover increased significantly with elevational distance, whereas nestedness decreased with elevational distance in the taxonomic and functional dimensions and showed no significant relationship in the phylogenetic dimension. Collectively, these results indicate a clear distance–decay pattern for total dissimilarity and turnover in small mammal communities of the western Tianshan Mountains (Soininen et al. 2007).

Increasing taxonomic and functional dissimilarity with elevational separation implies progressive divergence in both species composition and trait composition across the gradient. This pattern likely reflects limited dispersal across topographic barriers combined with species‐specific responses to spatially structured environmental variation, including environmental filtering and potential evolutionary constraints (da Silva et al. 2014). Given the relatively low dispersal capacity and high environmental sensitivity of small mammals, individuals may fail to track suitable habitats as conditions become harsher, increasing the likelihood of local extinctions and producing increasingly distinct communities at larger elevational distances (Varzinczak et al. 2019; Du et al. 2021).

By contrast, nestedness showed no consistent distance–decay signal across dimensions. The negative association between nestedness and elevational distance in the taxonomic and functional dimensions indicates weaker nestedness and less pronounced subset structure at greater elevational separation. Similar patterns have been reported in bats, rodents, marsupials, and bird assemblages (Varzinczak et al. 2019; Du et al. 2021). One interpretation is that the gain or loss of range‐restricted species or specific trait combinations can generate subset structure, such that geographically distant but species‐poor communities are nested within the species pools of richer communities (Ding et al. 2024).

### 4.4 Community Assembly Processes

Understanding how communities assemble remains a central objective in ecology (Kraft et al. 2011). We evaluated assembly processes along the elevational gradient by comparing observed multifaceted beta diversity with null expectations under random assembly. Across taxonomic, functional, and phylogenetic dimensions, SES patterns for total dissimilarity and turnover shifted from clustered to overdispersed with increasing elevational distance (Figure 5). At the same time, a substantial proportion of SES values fell within the null envelope (−1.96 to 1.96) (Figure 5), indicating that stochasticity is widespread in this system (Du et al. 2021; Wang et al. 2022; Ding et al. 2024). However, these results do not support a purely neutral interpretation; rather, they suggest assembly governed by the joint and scale‐dependent influence of stochastic dynamics and shifting deterministic processes (Du et al. 2021).

Specifically, SES values for total dissimilarity and turnover increased with elevational distance across all three dimensions, consistent with a weakening of environmental filtering and a strengthening of limiting similarity with increasing elevational separation. This directional shift has been reported across diverse taxa (Bishop et al. 2015; Du et al. 2021; Wang et al. 2022; Ding et al. 2024). For nestedness, patterns differed among dimensions. Taxonomic nestedness was largely indistinguishable from null expectations (SES between −1.96 and 1.96), suggesting a dominant role of stochasticity for this component. Functional nestedness exhibited a clearer decreasing trend (Figure 5c), indicating increasing clustering consistent with stronger environmental filtering and weaker limiting similarity. Phylogenetic nestedness showed no clear elevational trend and, similar to taxonomic nestedness, primarily reflected stochastic assembly.

Finally, the fitted relationships suggest that environmental filtering and limiting similarity may approach an equilibrium at intermediate elevational distances (Figure 5). This may contribute to the high proportion of SES values within the null envelope and suggests that the observed stochastic signal could arise from interactions between counterbalancing deterministic processes and neutral dynamics (Helmus et al. 2007; Du et al. 2021). As elevational distance among communities increases, environmental contrast and isolation intensify, strengthening species sorting and dispersal limitation (Varzinczak et al. 2019). In addition, competition for limited resources and microhabitat availability among small mammal functional groups may increase with elevational separation, further amplifying the signature of limiting similarity (Kelt et al. 1995; Du et al. 2021).

## 5 Conclusions

Exploring multifaceted beta diversity and its turnover and nestedness components is essential for elucidating the drivers of community assembly. Here, we compared taxonomic, functional, and phylogenetic beta diversity of small mammal communities across environmental gradients in the arid montane system of the western Tianshan Mountains, China, and inferred the associated assembly processes.

Turnover predominated across all three dimensions. Pairwise decompositions showed a consistent ordering of dissimilarity, with functional dissimilarity highest and phylogenetic dissimilarity lowest (β‐Funcsor/sim/sne > β‐Taxsor/sim/sne > β‐Phylosor/sim/sne). Hierarchical partitioning further identified elevational distance as the most influential predictor of multifaceted beta diversity. In addition, total dissimilarity and turnover exhibited clear distance–decay relationships with elevational distance. Null‐model analyses suggest that community assembly is shaped by a synergistic interaction between deterministic and neutral processes: with increasing elevational distance, the influence of environmental filtering weakened, whereas the influence of limiting similarity strengthened. Collectively, these results improve understanding of the processes shaping beta diversity in arid montane ecosystems and provide a theoretical basis for systematic biodiversity conservation in the mountain systems of Central Asia.

## Author Contributions

**Xiaohan Wang:** conceptualization (equal), data curation (lead), formal analysis (lead), software (lead), writing – original draft (lead). **Wenyu Song:** data curation (supporting), writing – review and editing (equal). **Yawen Xing:** data curation (supporting), writing – review and editing (supporting). **Yalan Wang:** data curation (supporting), writing – review and editing (supporting). **Xiaoxin Pei:** software (supporting), writing – review and editing (supporting). **Kenneth Otieno Onditi:** validation (supporting), writing – review and editing (supporting). **Hongjiao Wang:** data curation (supporting). **Quan Li:** validation (supporting). **Xueyou Li:** data curation (supporting). **Zhongzheng Chen:** conceptualization (equal), formal analysis (equal), funding acquisition (supporting), methodology (equal), project administration (equal), writing – original draft (equal). **Xuelong Jiang:** funding acquisition (lead), methodology (equal), project administration (equal).

## Funding

This research was funded by the Second Tibetan Plateau Scientific Expedition and Research (STEP) Program (Grant 2024QZKK0200) and the National Natural Science Foundation of China (Grant 32570523).

## Conflicts of Interest

The authors declare no conflicts of interest.

## Supporting information


**Table S1:** Locations of sampling sites, number of line transects (N), and main vegetation along the elevational gradient in the western Tianshan Mountains.
**Table S2:** The correlation between multifaceted beta diversity and environmental, geographical and elevational distances.
**Table S3:** Functional traits of each species and corresponding *K* and *p* values for each trait, western Tianshan Mountains.
**Table S4:** The first two axes and eigenvalues from the principal coordinate analysis (PCoA) of functional traits.
**Table S5:** Survey of small‐mammal species in the western Tianshan Mountains.
**Table S6:** Site‐wise species abundance matrix of small mammals across the western Tianshan Mountains.
**Table S7:** Species composition and elevational distribution of small mammals in the western Tianshan Mountains.
**Table S8:** Relationships between elevational distance and multidimensional beta diversity and SES (i.e., taxonomic, functional, and phylogenetic beta diversity) based on linear regression models.
**Table S9:** Independent contribution percentages of each explanatory distance matrix in the variations of multifaceted beta diversity.
**Table S10:** Variance inflation factors (VIF) of the retained predictor distance matrices.
**Figure S1:** Phylogenetic tree of small mammals in the western Tianshan Mountains.

## Data Availability

Raw data are provided as Supporting Information for the review process and will be deposited in a public data repository upon acceptance.
