# Supplementary material for: The Synergy Between Deterministic and Neutral Processes Drives Turnover‐Dominated Beta Diversity of Non‐Volant Small Mammals in the Western Tianshan Mountains, China
Source: Ecol Evol. 2026 Oct 30;16(11):e74538. doi: 10.1002/ece3.74538 (PMC13722604; doi:10.1002/ece3.74538)
Supplement: Supplementary file 1 [file ECE3-16-e74538-s001.docx]

Table S1 Locations of sampling sites, number of line transects (N), and main vegetation along the elevational gradient in the western Tianshan Mountains.

| Area | Line | Sampling sites | Vegetation | Longitude (°E) | Latitude (°N) | Elevation (m) |
| --- | --- | --- | --- | --- | --- | --- |
| 1 | 1 | Kurdening | 1、2 | 82.66 | 43.28 | 1000 |
|  | 2 | Kurdening | 1 | 82.88 | 43.26 | 1200 |
|  | 3 | Kurdening | 1 | 82.88 | 43.17 | 1500 |
|  | 4 | Kurdening | 1 | 82.89 | 43.15 | 1700 |
| 2 | 5 | Shata | 2 | 80.97 | 42.95 | 1600 |
|  | 6 | Shata | 1 | 80.55 | 42.69 | 1900 |
|  | 7 | Shata | 1、2 | 80.74 | 42.54 | 2300 |
|  | 8 | Shata | 1、2 | 80.84 | 42.46 | 2500 |
|  | 9 | Shata | 1、2 | 80.69 | 42.55 | 2800 |
| 3 | 10 | Tukai Desert | 3 | 80.86 | 43.95 | 500 |
|  | 11 | Tukai Desert | 3 | 80.74 | 43.93 | 550 |
|  | 12 | Tukai Desert | 3 | 80.74 | 43.94 | 550 |
| 4 | 13 | Ebinur Lake | 3 | 82.84 | 44.76 | 200 |
| 5 | 14 | Moso Bay Desert | 3 | 86.03 | 45.13 | 300 |
|  | 15 | Moso Bay Desert | 3 | 86.02 | 45.12 | 300 |

The vegetation types: 1 represents spruce forest; 2 represents herbaceous vegetation; 3 represents desert. Area 1: Kurdening, Gongliu County; Area 2: Shata, Zhaosu County; Area 3: Tukai Desert, Huocheng County; Area 4: Ebinur Lake, Jinghe County; Area 5: Moso Bay Desert, Manas County.

Table S2 The correlation between multifaceted beta diversity and environmental, geographical and elevational distances.

| **Beta diversity components** | **Distance matrices** | **Mantel r** | ***p*** |
| --- | --- | --- | --- |
| Taxonomic total dissimilarity | Geography | 0.39 | 0.004 |
| Taxonomic turnover | Geography | 0.42 | 0.002 |
| Taxonomic nestedness | Geography | -0.26 | 1.000 |
| Functional total dissimilarity | Geography | 0.40 | 0.001 |
| Functional turnover | Geography | 0.39 | 0.001 |
| Functional nestedness | Geography | -0.14 | 0.827 |
| Phylogenetic total dissimilarity | Geography | 0.33 | 0.003 |
| Phylogenetic turnover | Geography | 0.37 | 0.002 |
| Phylogenetic nestedness | Geography | -0.19 | 0.994 |
| Taxonomic total dissimilarity | Environment | 0.60 | 0.001 |
| Taxonomic turnover | Environment | 0.55 | 0.001 |
| Taxonomic nestedness | Environment | -0.11 | 0.749 |
| Functional total dissimilarity | Environment | 0.43 | 0.002 |
| Functional turnover | Environment | 0.49 | 0.001 |
| Functional nestedness | Environment | -0.24 | 0.96 |
| Phylogenetic total dissimilarity | Environment | 0.51 | 0.001 |
| Phylogenetic turnover | Environment | 0.54 | 0.001 |
| Phylogenetic nestedness | Environment | -0.16 | 0.862 |
| Taxonomic total dissimilarity | Elevation | 0.88 | 0.001 |
| Taxonomic turnover | Elevation | 0.89 | 0.001 |
| Taxonomic nestedness | Elevation | -0.42 | 1.000 |
| Functional total dissimilarity | Elevation | 0.65 | 0.001 |
| Functional turnover | Elevation | 0.72 | 0.001 |
| Functional nestedness | Elevation | -0.33 | 1.000 |
| Phylogenetic total dissimilarity | Elevation | 0.81 | 0.001 |
| Phylogenetic turnover | Elevation | 0.83 | 0.001 |
| Phylogenetic nestedness | Elevation | -0.18 | 0.987 |

Table S3 Functional traits of each species and corresponding K and p values for each trait, western Tianshan Mountains.

| **Species** | **W** | **HBL** | **TL** | **HFL** | **EL** |
| --- | --- | --- | --- | --- | --- |
| Eulipotyphla |  |  |  |  |  |
| Soricidae |  |  |  |  |  |
| *Sorex asper* | 8.23 | 69.75 | 41.92 | 12.96 | 7.43 |
| *Sorex minutus* | 3.52 | 53.06 | 36.00 | 9.98 | 5.38 |
| Rodentia |  |  |  |  |  |
| Sicistidae |  |  |  |  |  |
| *Sicista subtilis* | 7.52 | 64.00 | 70.60 | 15.00 | 11.60 |
| *Sicista tianshanica* | 9.74 | 71.47 | 110.87 | 17.79 | 14.87 |
| Dipodidae |  |  |  |  |  |
| *Dipus sagitta* | 37.85 | 101.25 | 146.50 | 58.75 | 18.50 |
| Muridae |  |  |  |  |  |
| *Meriones libycus* | 73.29 | 124.81 | 128.70 | 33.81 | 16.81 |
| *Meriones meridianus* | 41.44 | 106.12 | 108.38 | 29.17 | 14.98 |
| *Meriones tamariscinus* | 86.25 | 137.69 | 129.62 | 36.15 | 19.38 |
| *Rhombomys opimus* | 80.80 | 128.75 | 110.50 | 34.50 | 12.50 |
| *Apodemus uralensis* | 20.34 | 90.57 | 83.11 | 19.64 | 14.91 |
| *Rattus norvegicus* | 91.60 | 155.00 | 138.00 | 31.00 | 18.50 |
| Cricetidae |  |  |  |  |  |
| *Mus musculus* | 17.53 | 80.77 | 60.31 | 16.08 | 13.31 |
| *Lasiopodomys gregalis* | 24.36 | 97.75 | 30.50 | 15.95 | 11.47 |
| *Microtus arvalis* | 24.44 | 97.04 | 34.28 | 15.92 | 11.00 |
| *Alticola argentatus* | 26.99 | 100.38 | 41.88 | 17.38 | 16.25 |
| *Myodes centralis* | 25.82 | 101.11 | 47.88 | 17.27 | 13.72 |
| *Nothocricetulus migratorius* | 28.35 | 102.50 | 29.67 | 16.17 | 17.50 |
| Gliridae |  |  |  |  |  |
| *Dryomys nitedula* | 34.35 | 103.75 | 98.25 | 22.30 | 15.89 |
| Sciuridae |  |  |  |  |  |
| *Sciurus vulgaris* | 340.50 | 210.00 | 205.00 | 60.00 | 38.00 |
| Lagomorpha |  |  |  |  |  |
| Ochotonidae |  |  |  |  |  |
| *Ochotona macrotis* | 142.60 | 200.00 | 0.00 | 75.00 | 28.00 |
| K value | 1.04 | 0.78 | 0.71 | 1.12 | 0.94 |
| p value | 0.002 | 0.002 | 0.001 | 0.001 | 0.001 |

W: weight; HBL: head and body length; TL: tail length; HFL: hind foot length; EL: ear length.

Table S4 The first two axes and eigenvalues from the principal coordinate analysis (PCoA) of functional traits.

| **Species** | **traits1** | **traits2** |
| --- | --- | --- |
| *Sorex asper* | -0.728 | -0.104 |
| *Sorex minutus* | -0.904 | -0.035 |
| *Sicista subtilis* | -0.566 | 0.282 |
| *Sicista tianshanica* | -0.330 | 0.767 |
| *Dipus sagitta* | 0.411 | 0.611 |
| *Meriones libycus* | 0.283 | 0.526 |
| *Meriones meridianus* | -0.006 | 0.401 |
| *Meriones tamariscinus* | 0.458 | 0.406 |
| *Rhombomys opimus* | 0.173 | 0.173 |
| *Apodemus uralensis* | -0.267 | 0.220 |
| *Rattus norvegicus* | 0.493 | 0.504 |
| *Mus musculus* | -0.440 | -0.003 |
| *Lasiopodomys gregalis* | -0.466 | -0.521 |
| *Microtus arvalis* | -0.472 | -0.454 |
| *Alticola argentatus* | -0.288 | -0.444 |
| *Myodes centralis* | -0.340 | -0.325 |
| *Nothocricetulus migratorius* | -0.282 | -0.641 |
| *Dryomys nitedula* | -0.095 | 0.330 |
| *Sciurus vulgaris* | 2.222 | 0.648 |
| *Ochotona macrotis* | 1.145 | -2.448 |
| Eigenvalue | 3.822 | 0.723 |
| Relative eigenvalue (%) | 0.764 | 0.145 |
| Cumulative eigenvalue (%) | 0.764 | 0.909 |

Table S5 Survey of small-mammal species in the western Tianshan Mountains.

| **Taxon** | **Species** | **Individual** | **Rate** |
| --- | --- | --- | --- |
| Eulipotyphla |  |  |  |
| Soricidae |  |  |  |
| Sorex | Sorex asper | 25 | 4.10% |
|  | Sorex minutus | 53 | 8.69% |
| Rodentia |  |  |  |
| Sicistidae |  |  |  |
| Sicista | Sicista subtilis | 5 | 0.82% |
|  | Sicista tianshanica | 39 | 6.39% |
| Dipodidae |  |  |  |
| Dipus | Dipus sagitta | 4 | 0.66% |
| Muridae |  |  |  |
| Meriones | Meriones libycus | 27 | 4.43% |
|  | Meriones meridianus | 86 | 14.10% |
|  | Meriones tamariscinus | 13 | 2.13% |
| Rhombomys | *Rhombomys opimus* | 4 | 0.66% |
| Apodemus | Apodemus uralensis | 169 | 27.70% |
| Rattus | Rattus norvegicus | 2 | 0.33% |
| Mus | Mus musculus | 13 | 2.13% |
| Cricetidae |  |  |  |
| Lasiopodomys | Lasiopodomys gregalis | 20 | 3.28% |
| Microtus | Microtus arvalis | 25 | 4.10% |
| Alticola | Alticola argentatus | 8 | 1.31% |
| Myodes | Myodes centralis | 89 | 14.59% |
| *Nothocricetulus* | *Nothocricetulus* migratorius | 6 | 0.98% |
| Gliridae |  |  |  |
| Dryomys | Dryomys nitedula | 20 | 3.28% |
| Sciuridae |  |  |  |
| Sciurus | Sciurus vulgaris | 1 | 0.16% |
| Lagomorpha |  |  |  |
| Ochotonidae |  |  |  |
| Ochotona | Ochotona macrotis | 1 | 0.16% |

Table S6 Site-wise species abundance matrix of small mammals across the western Tianshan Mountains.

| **Species / Sampling Site** | **Kurdening** | **Shata** | **Tukai Desert** | **Ebinur Lake** | **Moso Bay Desert** |
| --- | --- | --- | --- | --- | --- |
| *Sorex asper* | 7 | 18 | 0 | 0 | 0 |
| *Sorex minutus* | 24 | 29 | 0 | 0 | 0 |
| *Sicista subtilis* | 5 | 0 | 0 | 0 | 0 |
| *Sicista tianshanica* | 37 | 2 | 0 | 0 | 0 |
| *Dipus sagitta* | 0 | 0 | 0 | 0 | 4 |
| *Meriones libycus* | 0 | 0 | 0 | 2 | 25 |
| *Meriones meridianus* | 0 | 0 | 21 | 20 | 45 |
| *Meriones tamariscinus* | 0 | 0 | 7 | 6 | 0 |
| *Rhombomys opimus* | 0 | 0 | 0 | 0 | 4 |
| *Apodemus uralensis* | 74 | 45 | 43 | 4 | 3 |
| *Rattus norvegicus* | 0 | 0 | 2 | 0 | 0 |
| *Mus musculus* | 0 | 2 | 7 | 1 | 3 |
| *Lasiopodomys gregalis* | 0 | 20 | 0 | 0 | 0 |
| *Microtus arvalis* | 25 | 0 | 0 | 0 | 0 |
| *Alticola argentatus* | 0 | 8 | 0 | 0 | 0 |
| *Myodes centralis* | 39 | 50 | 0 | 0 | 0 |
| *Nothocricetulus migratorius* | 2 | 0 | 4 | 0 | 0 |
| *Dryomys nitedula* | 16 | 0 | 1 | 3 | 0 |
| *Sciurus vulgaris* | 1 | 0 | 0 | 0 | 0 |
| *Ochotona macrotis* | 0 | 1 | 0 | 0 | 0 |
| *Total individuals per site* | 230 | 175 | 85 | 36 | 84 |

Table S7 Species composition and elevational distribution of small mammals in the western Tianshan Mountains.

| **Species** | **200** | **300** | **300** | **500** | **550** | **550** | **1000** | **1200** | **1500** | **1600** | **1700** | **1900** | **2300** | **2500** | **2800** |
| --- | --- | --- | --- | --- | --- | --- | --- | --- | --- | --- | --- | --- | --- | --- | --- |
| *Sorex asper* |  |  |  |  |  |  |  |  | + | + | + | + | + | + | + |
| *Sorex minutus* |  |  |  |  |  |  |  |  | + | + | + | + | + | + | + |
| *Sicista subtilis* |  |  |  |  |  |  |  |  | + | + | + |  |  |  |  |
| *Sicista tianshanica* |  |  |  |  |  |  |  |  | + | + | + | + | + |  |  |
| *Dipus sagitta* |  | + | + |  |  |  |  |  |  |  |  |  |  |  |  |
| *Meriones libycus* | + | + | + |  |  |  |  |  |  |  |  |  |  |  |  |
| *Meriones meridianus* | + | + | + | + | + | + |  |  |  |  |  |  |  |  |  |
| *Meriones tamariscinus* | + | + | + | + | + | + |  |  |  |  |  |  |  |  |  |
| *Rhombomys opimus* |  | + |  |  |  |  |  |  |  |  |  |  |  |  |  |
| *Apodemus uralensis* | + | + | + | + | + | + | + | + | + | + | + | + | + | + | + |
| *Rattus norvegicus* |  |  |  |  | + | + |  |  |  |  |  |  |  |  |  |
| *Mus musculus* | + | + | + | + | + | + | + | + | + | + | + | + |  |  |  |
| *Lasiopodomys gregalis* |  |  |  |  |  |  |  |  |  | + | + | + | + | + | + |
| *Microtus arvalis* |  |  |  |  |  |  |  |  | + | + | + |  |  |  |  |
| *Alticola argentatus* |  |  |  |  |  |  |  |  |  |  |  |  |  | + | + |
| *Myodes centralis* |  |  |  |  |  |  |  | + | + | + | + | + | + | + | + |
| *Nothocricetulus migratorius* |  |  |  | + | + | + | + |  |  |  |  |  |  |  |  |
| *Dryomys nitedula* | + | + | + | + | + | + | + | + | + |  |  |  |  |  |  |
| *Sciurus vulgaris* |  |  |  |  |  |  |  |  | + |  |  |  |  |  |  |
| *Ochotona macrotis* |  |  |  |  |  |  |  |  |  |  |  |  |  | + |  |

+ indicated that the species occurred in the elevational band.

Table S8 Relationships between elevational distance and multidimensional beta diversity and SES (i.e., taxonomic, functional, and phylogenetic beta diversity) based on linear regression models.

| **Beta diversity components** | **Intercept** | **Slope** | **R²** | ***p*** |
| --- | --- | --- | --- | --- |
| Taxonomic total dissimilarity | 0.17 | 3.42e-04 | 0.77 | < 0.001 |
| Taxonomic turnover | 0.027 | 3.96e-04 | 0.79 | < 0.001 |
| Taxonomic nestedness | 0.143 | -5.37e-05 | 0.16 | < 0.001 |
| Phylogenetic total dissimilarity | 0.109 | 2.68e-04 | 0.65 | < 0.001 |
| Phylogenetic turnover | 8.62e-03 | 2.86e-04 | 0.68 | < 0.001 |
| Phylogenetic nestedness | 0.09 | -1.81e-05 | 0.02 | 0.080 |
| Functional total dissimilarity | 0.403 | 2.73e-04 | 0.42 | < 0.001 |
| Functional turnover | 0.046 | 3.93e-04 | 0.50 | < 0.001 |
| Functional nestedness | 0.356 | -1.21e-04 | 0.10 | < 0.001 |
| SES of Taxonomic total dissimilarity | -2.54 | 2.49e-03 | 0.75 | < 0.001 |
| SES of Taxonomic turnover | -2.57 | 2.41e-03 | 0.75 | < 0.001 |
| SES of Taxonomic nestedness | 2.52 | -2.54e-03 | 0.72 | < 0.001 |
| SES of Phylogenetic total dissimilarity | -1.65 | 2.04e-03 | 0.61 | < 0.001 |
| SES of Phylogenetic turnover | -1.45 | 2.01e-03 | 0.63 | < 0.001 |
| SES of Phylogenetic nestedness | -0.246 | 1.14e-04 | 0.01 | 0.327 |
| SES of Functional total dissimilarity | -0.91 | 1.44e-03 | 0.43 | < 0.001 |
| SES of Functional turnover | -0.938 | 1.62e-03 | 0.43 | < 0.001 |
| SES of Functional nestedness | 0.254 | -4.94e-04 | 0.09 | 0.001 |

Table S9 Independent contribution percentages of each explanatory distance matrix in the variations of multifaceted beta diversity.

|  | AP | NDVI | HD | HH | GEO | ELE |
| --- | --- | --- | --- | --- | --- | --- |
| **Taxonomic** |  |  |  |  |  |  |
| total dissimilarity | 17.82 | 13.28 | 1.09 | 0.18 | 9.57 | 58.07 |
| turnover | 20.14 | 13.41 | 0.22 | 0.23 | 8.32 | 57.68 |
| nestedness | 27.46 | 7.32 | 29.70 | 1.75 | 4.57 | 29.20 |
| **Functional** |  |  |  |  |  |  |
| total dissimilarity | 16.74 | 21.30 | 1.92 | 1.99 | 15.63 | 42.42 |
| turnover | 25.28 | 15.20 | 0.42 | 0.32 | 8.94 | 49.86 |
| nestedness | 34.89 | 2.56 | 1.96 | 15.59 | 7.48 | 37.53 |
| **Phylogenetic** |  |  |  |  |  |  |
| total dissimilarity | 19.75 | 15.28 | 0.46 | 0.43 | 7.14 | 56.94 |
| turnover | 22.81 | 13.30 | 0.27 | 0.15 | 7.36 | 56.11 |
| nestedness | 49.91 | 2.09 | 3.91 | 20.22 | 5.81 | 18.06 |

AP, annual precipitation; NDVI, normalized difference vegetation index; HD, human disturbance; HH, habitat heterogeneity; GEO, geographical distance; ELE, elevational distance.

The independent contribution percentage of each environmental distance matrix was determined using hierarchical partitioning analysis following multiple regression on distance matrices.

Table S10 Variance inflation factors (VIF) of the retained predictor distance matrices.

| Predictor | VIF |
| --- | --- |
| AP | 3.01 |
|  | 1.24 |
| HD | 1.18 |
| HH | 1.18 |
| GEO | 1.61 |
| ELE | 2.68 |

FIGURE S1. Phylogenetic tree of small mammals in the western Tianshan Mountains.
